# 

**Published:** 1881-10-20

**Authors:** 


### TABLE OF CONTENTS.

ORIGINAL AND SELECTED ARTICLES.

The Nature, Pathology and Treatment of Dipsomania, by Edward C. Mann, M. D., of New York, 361. Phagomania, by L. G. Hardman, M. D., of Georgia, 367. A Case of Malposition of the Fœtus sixty hours in labor, by Wm. T. Beall, M. D., of Miss., 368. A Case of Facial Paralysis and Lumbago, by C. H. Wagner, M. D., of Waterford, Mississippi, 369 Abstracts of Papers read at the International Medical Congress, 370. Diphtheritic Croup, by J. M. Batten, M. D., of Pittsburg, Penn., 373. Typho-Malarial Fever, by A. C. Osterman, M. D., 374. Skin-Grafting in Loss of Scalp, by William L. Bradley, M. D., of New Haven, Conn., 376.

ABSTRACTS AND GLEANINGS.

Otorrhœa, 378. The Use of Quebracho in Dyspnœa, 380. Aspiration of the Gall-Bladder, 381. The Treatment of Ranula; Treatment of Chorea, 382. Medical Thermometry, 383. Radical Treatment of Hydrocele by Injection of Carbolic Acid; Two Yards of Intestine Removed, 384. Treatment of Eczema of the Hand, 385. Aromatized Glycerine and its Uses, 386. Enlarged Spleen; A Resurrection; Prizes Worth Striving for, 387. A Case of Prolonged Somolence; Morbus Coxarius Treated by the Physiological Method; Ergot in Tuberculosis, 388. The Treatment of Erysipelas; Common Bread Poultice; The Reported Death of Dr. Tanner; Dangers of Tents, 389. Purgatives by Hypodermic Injection; Fish Bones in Pharynx; For a Dinner Pill; Gonorrhœa, 390.

SCIENTIFIC ITEMS.

New Electrical Devices in Engine Houses; Balloons in Submarine Work; How Long Should We Sleep, 391. The Painlessness of Death; Rendering Cotton Goods Uninflamable, 392.

PRACTICAL NOTES AND FORMULÆ.

Dry Catarrh; Painful Micturition; Anti-emetic; Iodide of Potassium in Typhoid Fever, 393. Hemorrhoids; C. O. D.; Luton's Exhilerant Mixture, 394. Tape Worm; Radical Cure for Rupture; Cure of Goiter by Fluoric Acid; Chronic Ulcers; Pruritus Vulvæ, 395.

EDITORIALS AND MISCELLANEOUS.

Sharp & Dohme; Kilner's Druggist Formulary; Southern Medical College; Unfortunate; Alumist and Neurologist; New Journals; American Public Health Association, 396. Mississippi State Medical Association, 397. Interesting Trade-Mark Litigation; Book Notices, 398. Special Notices, 400.

